# Strengthening social capital in the Sri Lankan population: A qualitative exploration of factors driving the mothers’ support groups initiative during economic crisis

**DOI:** 10.34172/hpp.2023.35

**Published:** 2023-12-16

**Authors:** Millawage Supun Dilara Wijesinghe, Upeksha Gayani Karawita, Nissanka Achchi Kankanamalage Ayoma Iroshanee Nissanka, Balangoda Muhamdiramlage Indika Gunawardana, Weerasinghe Mudiyanselage Prasad Chathuranga Weerasinghe, Vithanage Chandima Nayani Vithana, Kanchana Lanka Kumari Mahagamage, Singappuli Arachchilage Sanjeewanie Champika Karunaratne, Ranjith Batuwanthudawe

**Affiliations:** Health Promotion Bureau, Sri Lanka

**Keywords:** Social capital, Nutritional status, Community participation

## Abstract

**Background:**

Social capital is a concept that has been identified to improve health outcomes in many populations. Due to COVID-19 and many other factors, Sri Lanka faced a massive economic crisis that affected the nutrition of communities. Many community engagement initiatives have begun to promote the country’s nutrition during the worst-hit years. The Mothers’ Support Groups initiative is one of the existing community engagement initiatives that is well known for strengthening community social capital. This article discusses how the Mothers’ Support Groups (MSG) initiative in Sri Lanka contributed to improving social capital in Sri Lanka during the economic crisis, focusing on nutrition.

**Methods:**

We conducted a case study on the activities undertaken by mothers’ support groups in view of how they focused on social capital. We selected all activities presented by districts that improved social capital related to nutrition promotion captured in the YouTube video stream. We analyzed these qualitative data to identify the main themes related to social capital and nutritional promotion. Two coders transcribed the video recordings. We analyzed the data using the iterative thematic inquiry (ITI) method and initially assessed beliefs about concepts, building new beliefs through encounters with data, listing tentative themes, and evaluating themes through coding.

**Results:**

Six major themes were identified (that social capital had been strengthened to promote nutrition): awareness creation of nutrition, home gardening promotion, promoting livestock farming, minimizing food waste, improving the home economy, and psychosocial health promotion. The most common forms of social capital encountered in these themes were bonding, bridging, and linking. Furthermore, strengthening structural social capital is more prominent than strengthening cognitive social capital.

**Conclusion:**

Social capital can improve nutritional status during crises. Activities that can be used to achieve this vary from simple awareness creation among communities to more advanced psychosocial health promotion. Overall, social capital contributed to the community development aspect of health promotion to a greater extent.

## Introduction

 The emergence of the COVID-19 pandemic in 2019-2022 has resulted in a global economic crisis. Due to the pandemic, the economies of many countries were affected, with South Asia being the hardest hit.^1^ Sri Lanka was also affected by the pandemic from 2020 onwards. Due to the country’s declining foreign reserves following the pandemic, Sri Lanka experienced a severe economic crisis. As a result, there were shortages of essential goods such as food, fuel, medicine, cement, and other supplies.^2^ This pushed over 30% of the Sri Lankan population to food insecurity, leading to reduced meals, skipping meals, and more than 60% of families eating less nutritious food.^3^ Low-income populations’ nutritional condition and health are predicted to be negatively impacted by the food price issue over the long term, with young children in preschool and infants being particularly at risk. Studies have also shown that caloric intake and diet quality are greatly affected during economic recessions, especially among disadvantaged populations.^4^ Many interventions have been implemented to improve nutrition to overcome the financial crisis in Sri Lanka. Livelihood assistance programs targeting small holder farmers, immediate food and nutrition assistance programs, and cash-based assistance programs for at-risk communities were some of them.^3^

 Furthermore, as in many communities, there is a lack of nutrition awareness among Sri Lankan communities.^5^ This nutrition deficiency affects several aspects, including poor dietary habits, lack of knowledge on a balanced diet, and underestimation of the importance of having a regular good nutritious diet.^6^ The poor dietary habits among the community can be influenced by their cultural norms due to the availability and accessibility issues for nutritious foods.^7^ and misunderstanding about the nutritional recommended requirement within the families. Such gaps in awareness can have serious consequences for the health and well-being of the community, especially among vulnerable groups of pregnant mothers and children during this economic downturn.

 Social capital, in its earliest definitions, refers to the things that people value most in life, such as goodwill, mutual trust and friendship, sympathy, and social interaction among the individuals and families who make up a social unit.^8^ With regard to communities and in its border context, it can be used to refer to the social cohesion and investment of individuals in communities.^9^ Putnam revisited the concept of social capital in his book at the turn of the millennium, highlighting how individuals can become disconnected from families, neighbors, and communities, leading to many adverse impacts.^10^

 Individuals engage in various activities that affect their use of financial, physical, and human capital. People form groups based on their residence, community, and other factors. These groups play a role in shaping individual identity, norms, beliefs, and priorities.^11^ Through these social networks, people share information, provide support, and collaborate to achieve collective goals that may not be attainable by individuals working alone. This mutual relation and recognition among individuals within these groups creates “social capital,” which refers to the available resources that people can use for productive activities.^12^

 The essence of social capital deals with the nonmonetary resources generated through networking with others, fostering a sense of belonging, trust, and influence in communities.^9^ There is ample evidence to suggest that there is a positive relationship between social capital and health.^13^

 Social cohesion and social network approaches to health are traditionally suggested approaches to understanding how social capital predicts better health outcomes.^14^ However, in addition to cohesiveness and network approaches, there are other approaches that can be used to understand the relationship between social capital and health. One approach categorizes social capital in three ways: dimension, setting, and level. The dimensions include cognitive, structural, bonding, bridging, and linking subdomains. Settings include the places where it occurs, and levels include whether it occurs at the individual or group level.^13^

 As described above, social capital is an attribute related to communities.^9^ Therefore, by engaging and strengthening the capacity of the community, social capital can be improved, leading to better health outcomes. Hence, any community engagement initiative to improve social capital in a given setting at the group level should focus on strengthening the key dimensions, such as bonding, bridging, and linking.^13^ Bonding can connect individuals of similar identities and values (social connections between similar individuals), and bridging spans social differences such as connections between socially dissimilar individuals.^15,16^ Linking refers to connecting individuals and groups in communities within hierarchies of power and authority.^14^

 Sri Lanka’s Health Promotion Bureau also mobilized its community engagement platform, the Mothers’ Support Groups (MSG) initiative, throughout the island to strengthen social capital in communities to face the nutritional crisis fueled by the economic downturn. Social capital has been shown to encourage better nutrition during crises,^17^ and to protect against food insecurity^18^ at the community level through mechanisms such as knowledge sharing and product sharing, which facilitate food availability and access to food.^19^ It has also been reported that improving social capital will buffer economic and health shocks and improve child health status.^20^ Although the Mothers’ Support Groups community engagement platform has been working at the community level to improve social capital to promote nutrition for over a year, there has been no pragmatic assessment of these initiatives.. The primary objective was to examine how communities understand and utilize the different dimensions of social capital–bonding, bridging, and linking–to improve the nutritional status of the community. Understanding these dimensions is necessary to evaluate the effectiveness of social capital in promoting collaboration, trust, and mutual support among community members. Furthermore, this research examines how social connections are put into action within the community. By looking at real-life examples and projects in the community, we want to determine how social connections are used to achieve common goals, especially in regard to nutrition issues during times of economic crisis.

## Materials and Methods

###  Gathering evidence for strengthening social capital

 We conducted a case study of activities carried out by MSG in Sri Lanka to improve social capital during the economic crisis, focusing on nutrition promotion. The exercise was exploratory in nature, and the analysis unit was a single-mother support group from each district.

 The recorded video streams from the National Review of Mothers’ Support Groups on December 13 and 14, 2022, organized and conducted by the Health Promotion Bureau, are publicly available on the official YouTube site.^21,22^ The case study consisted of individual presentations by public health midwives (PHMs) from 25 administrative districts of the country (each district was free to select the best MSG in its district and present its selected group activities at the national level). In addition to the district presentations, the Colombo Municipal Council (CMC) and the National Institute of Health Sciences (NIHS) were presented during the review (separate health administrative areas in the two districts). These presentations, ranging from seven to 14 minutes (median duration of 11 minutes), provided valuable insights into the utilization of social capital within the group. According to the initial guidelines sent before the presentation, only nutritional promotion activities were entertained. Therefore, we selected all activities presented by districts that improved social capital related to nutrition promotion that was captured in the YouTube video stream and served as the data source. These presentations provided qualitative data that were thematically examined to identify the main themes related to social capital and nutritional promotion.

 This exercise can be categorized under program evaluation, falling within organizational requirements, to be used for assessment and improvement purposes with a negligible risk that involves the use of existing collections of publicly available data or records that contain only nonidentifiable data about activities conducted at the community level. Informed consent was obtained from each district’s participant team for further analysis of the content presented. We did not use any specific research protocols or guidelines.

###  Thematic analysis to identify strengthening social capital to promote nutrition

 Two coders transcribed the video recordings using MS Word on March 2023. The data were analyzed by a team comprising four registrars in community medicine who were trained in qualitative data analysis. They read and reread the transcribed video recordings to familiarize themselves with the content. No special computer software was used for analysis. To minimize bias, we did not quantify the interpretations of the respondents, and the researchers noted their own influence on the data. The qualitative data analysis process was data-driven and inductive. Thematic analysis was performed using pattern recognition, with emerging themes becoming categories for analysis. We analyzed the data using the iterative thematic inquiry (ITI) method described by Morgan and Nica.^23^ According to the ITI method, we initially assessed beliefs of concept, building new beliefs through encounters with data, listing tentative themes, and evaluating themes through coding. This method focuses on themes from the beginning and continues throughout; therefore, coding in ITI occurs at a late stage in the analysis process when it compares a tentative summary of the initial process to the original data.^23^ We assigned codes to segments of the data related to specific themes. Similar codes were grouped together to identify broader themes.

 Understanding the dimension of social capital is important for coding. The dimensions of social capital vary according to its definition. The most widely used dimensions include structural (configuration and pattern of social relationships, including structures of social organization), relational (characteristics and qualities of social relationships), and cognitive (shared understanding that provides systems of meaning).^24^ A more elaborate definition of dimensions was provided by Ehsan et al,^13^ which categorized it into cognitive (perception of trust, reciprocity, norms, and values within a community), structural (quantity of relationships and membership in institutions that can bring individuals and groups together), bonding (social resources that an individual can obtain through close networks or groups with similar sociodemographic characteristics), bridging (resources that can be accessed from networks or groups with different characteristics), and linking (connections with individuals and groups within hierarchies of power and authority). Furthermore, a more simplified classification of dimensions was identified by the World Bank’s social capital implementation framework,^25^ which includes groups and networks, trust and solidarity, collective action and cooperation, social cohesion and inclusion, and information and communication. The dimensions of social capital strengthened for each identified theme were coded separately, thus falling within the above definitions.

 The finalized themes were reviewed for accuracy, missing areas, and alternative interpretations.^23^ The methodology explored social capital within mothers’ support groups by analyzing data from district presentations during the annual review. These presentations reveal how social capital was employed, including building networks, mobilizing resources, fostering collaboration, and promoting collective action.

 By employing this methodology, this study aimed to gain a comprehensive understanding of how social capital was effectively utilized by different districts by mothers’ support groups. The analysis of data collected from district presentations provided insights into the specific ways in which social capital was employed, such as by building networks and mobilizing resources. Thematic analysis identified recurring patterns, revealing common strategies, challenges, and successes associated with effective utilization of social capital (Table 1). This research contributes to the understanding of the key factors and practices that contribute to achieving the objectives of mothers’ support groups.

**Table 1:** Main themes identified and their brief explanations

**Theme**	**Brief explanation of the theme**
Awareness creation of nutrition	Increase nutrition knowledge in the village environment using the concept of social capital
Home gardening promotion	Promotion of home gardening and the improvement of the well-being of communities using the concept of social capital
Promote livestock farming	Empowerment of small-scale livestock farmers in the communities using social capital
Minimize food wastage	Reducing food waste by using social capital as the basis and enticing people to change their habits for environmentally friendly practices using social capital
Improve home economy	Encourage self-employment by various mean using social capital.
Psychosocial health promotion	Promoting social connections, support, and a sense of belonging within communities using concepts of social capital

## Results: Identified themes and how social capital was strengthened

###  Awareness creation of nutrition

 Social capital promotes nutrition-related health behaviors through various forms of social support and social networks within the community.^26^ The MSG members carried out several initiatives to increase nutrition knowledge in the village environment using the concept of social capital.

 Within the realm of social capital, various activities have been undertaken to foster both the bonding and bridging dimensions. These activities encompassed raising awareness through village-based organizations, such as the Elderly, Children, and Youth Societies, which were created with the active participation of villagers. The Elderly Society provides a platform for senior citizens, while the Children’s and Youth Society acts as mentors and role models for the community’s younger individuals, discussing nutrition and sharing experiences related to healthy living, thus inspiring bonding social capital.

 Underweight children visiting the weighing post are asked to recall their food intake using the 24-hour dietary recall method, supervised by a public health nursing sister and a PHM. The objective of this activity is to assess the nutritional status of these children and identify any potential deficiencies or gaps in their food intake. Individuals and communities possess social capital in the form of resources and relationships that can be utilized to achieve common goals and enhance overall well-being. The involvement of healthcare professionals, such as public health nursing sisters and supervisory PHMs, exemplifies the linking concept of social capital.

 The nutrition committee, known as ‘Poshana Kamituwa,’ provides a platform for community members to discuss and take action on nutrition-related issues. This committee consists of various stakeholders, including community members, health professionals, village educators, and local leaders. The committee’s mission is to encourage the sharing of information, collaboration, and collective efforts to improve nutritional outcomes. Therefore, ‘Poshana Kamituwa’ serves as a platform for networking among committee members and healthcare workers, emphasizing the bridging and linking concept of social capital to address nutrition-related issues.

 In addition, the MSG members distributed pouches of nutritious food with the aid of local bank officers. They also conducted nutrition awareness programs with the assistance of agricultural officers, village religious leaders, and schoolteachers. Furthermore, with the aid of government and nongovernmental organizations, they conducted a food exhibition, printed a booklet with locally available nutritious food menus, and invited a nutritionist to conduct a nutrition awareness programme in the village and engage in other activities that fall under the linking concept of social capital.

 Moreover, they established a WhatsApp group for MSG members and a Facebook page for information sharing to raise awareness of nutrition as a component of the social capital network.

 By utilizing the power of social capital, they have also established a norm for weighing children on a specific day, introduced the “well pouch” (a bag containing healthy ingredients) and “ill pouch” (a bag containing unhealthy ingredients) concepts, and established a “nutrition calendar” as a norm creation under social capital for the majority of affected at-risk, vulnerable people in the village.

###  Home gardening promotion

 Home gardening is an important strategy for enhancing food security in the household, contributing to improved nutrition, health, and community well-being during an economic crisis.^27^ Similar to the above, the bonding social capital domain plays a significant role in the promotion of home gardening and the improvement of the well-being of communities.

 Bonding social capital was utilized by Mothers’ Support Groups to distribute gardening equipment, seeds, and nursery buds among community members; in addition, they shared their knowledge on organic pesticides and weedicides, which optimized the quality of home gardening products. This has promoted mutual support and sharing of resources and is vital for the success of home-gardening programs. Furthermore, they exchanged excess harvest among villages, which enhanced food security and nutrition by ensuring that the extra harvest from home gardens was distributed to those in need.

 MSGs have strengthened social capital by involving youth generations, such as school leavers (who have completed their final year of education at a school and are leaving the educational institution to transition to the next stage of their lives), in home-gardening programs. Furthermore, bridging social capital was exploited to promote home gardening through existing community groups such as elderly societies, women’s societies, and civil security committees. This has encouraged community ownership, participation, and sustainability of home-gardening programs.

 In the home gardening program context, several MSG activities strengthened linking social capital by promoting collaboration and knowledge sharing among different stakeholders to promote nutrition. For example, government officers such as Agricultural Officers (AO) in Divisional Secretariat Offices have supported home gardening by distributing seeds and nursery buds to farmers at a reasonable price and providing vertical cultivation named “Waga Kuluna”, “potted farming,” and mushroom gardens. Additionally, the AO has provided technical knowledge on composting and organic fertilizer preparation that has helped linking social capital by promoting sustainable gardening practices. Furthermore, several government officers provided financial support for home gardening to promote nutrition during the economic crisis.

 Additionally, the home garden program is facilitated by the participation of external stakeholders, such as government departments, UNICEF, and other NGOs that have provided technical guides, financial support, expertise, and networking opportunities.

 The concept of a “nutritious fence” is planting specific crops with nutritional value along the fence or boundary of the home garden. This innovative concept in home gardening programs has contributed to norm creation by promoting nutrition, food security, and sustainability.

###  Promote livestock farming

 In the context of promoting livestock farming, MSG members have received financial support from local donors who provide funding to establish a poultry farm, which creates bonding social capital. Furthermore, MSG members engaged in cattle farming and sold fresh milk at a low cost to other group members, which created mutual benefits among them as they supported each other’s livelihood. Evidence shows that the empowerment of small-scale livestock farmers is crucial for attaining sustainable agriculture. Furthermore, evidence also highlights attributes characterized by factors such as willingness, openness, motivation, and participation that significantly impact the empowerment linked to social capital.^28^

 Additionally, exchanging fresh milk at a low cost promoted affordability and access to nutritious food while ensuring food security among group members.

 Linking social capital strengthened livestock farmers by receiving technical and financial support for curd and yogurt production, which could be sold in the local market. Government officers at the Divisional Secretariat Office supported this. Additionally, MSG members were introduced to poultry farming in one household, and chicks were distributed among the most vulnerable households in the community with the help of the Samurdhi officer and the AO.

###  Minimize food wastage

 Reducing food waste is crucial for improving community nutrition by using social capital as the basis and enticing people to change their habits. Food producers, for instance, might embrace healthier diets that include more vegetables and environmentally friendly practices such as composting. They typically transferred food waste from their flats to Garden’s compost bins.^29^ With the assistance of agricultural officers and school agriculture teachers in the area, MSG members carried out a program to disseminate knowledge of food preservation techniques (bonding and linking social capital). In addition, they constructed waste disposal containers on either side of the main road by strengthening the bridging social capital.

 They obtained assistance from an advisor from the Agricultural Research Institute regarding food preservation techniques and how to enhance them as a form of self-employment to generate income as a strength by linking social capital.

###  Improve home economy

 All community members profit from synergy created by social capital. The MSG members began selling agricultural products to major government and nongovernment organizations, held fairs to sell the harvest from their own gardens (at weighing centers), and established a society called “Ithirum Samithiya” with the assistance of the leaders of the MSG. Furthermore, they encouraged self-employment, and selling these products was an activity under the dimension of bonding in social capital. Additionally, as a component of linking social capital, they received technical assistance and financial support for the beginning of self-employment from the region’s Economic Development Officer (EDO).

###  Psychosocial health promotion

 Social capital can positively impact psychosocial health by promoting social connections, support, and a sense of belonging within communities.^30^ “Play hour” play activities, which facilitate the environment for children to play with each other in some communities, foster bonding social capital. These play activities provide children with opportunities to build friendships that contribute to their psychosocial health. In addition, creating a special group called “Mithuru Sansadaya”, which includes husbands of MSG members, strengthened bonding social capital and promoted psychosocial health-related activities. This group provided a platform for husbands to share their experiences and support each other to promote health and well-being within the community.

 Life skills programs conducted by health education officers serve as a form of linking social capital that promotes psychosocial health among individuals. Social capital networking is a practical approach to promoting psychosocial health, particularly in the context of dengue control and prevention, filariasis awareness, and blood donation programs. Furthermore, health authorities and organizations collaborated with MSG to raise awareness.

 Certain social capital norms promote psychosocial health, such as “taking at least one meal together as a family”; “Happiness Calendar” (a special calendar made by children where days are marked with simile faces); and special savings called “Buddi Kataya” (a local savings scheme where children save money collectively and use it for good activities). These norms contribute to better psychosocial health among community members.

## Discussion

 Our case study identified six themes among mothers’ support groups that strengthened social capital and promoted nutrition in communities. The most common dimensions of social capital encountered were bonding, bridging, and linking. In addition, strengthening structural social capital is more prominent than strengthening cognitive social capital.

 People can access information through community networks. The potential for knowledge acquisition is created by network members through membership in the network and the resulting recurrent and long-term knowledge-exchange linkages. One of the most significant advantages of social capital is its access to new sources of knowledge. Additionally, evidence suggests that close social connections between organizational actors increase knowledge transfer.^31^ For example, our case study observed awareness creation and information sharing about nutritious foods among mothers’ support groups and the community. In addition, norm creation, or cognitive social capital, is an important domain of social capital that involves the development of shared values, beliefs, and practices within a community, which was also demonstrated by the mothers’ support group network.^14^

 Furthermore, evidence states that social capital improves children’s health by sharing health information and promoting the mother’s physical and mental well-being.^20^ This was demonstrated in our findings by introducing nutritious food menus to vulnerable groups such as young children under five years of age and pregnant women as a result of bonding social capital. Food exhibitions, nutrition awareness programs, and nutritious food menu sharing are examples of the smooth transfer of local knowledge among network members. This represents the uniqueness and strong qualities of social capital that strongly influence the knowledge transfer mechanisms in local communities.^31^ Moreover, mothers’ support groups promoted sharing food items and knowledge (through social media and nutrition committees) within the community, indicating how social capital helps increase food availability and access.^19^

 Evidence points to social capital as essential in connecting small producers and national and external parties in seed exchange connections. Furthermore, it is implied that social capital contributes to food availability by facilitating seed exchange and farm management practices.^19^ Our case study also demonstrated these seed exchange connections in mothers’ support groups. Moreover, norms and values of home gardening were also shared among mothers’ support groups, which is also highlighted by other authors.^20^ The reverse direction of the relationship also holds true in the case where home gardening is linked to social capital and food security. Home gardening not only produces food but also fosters social capital by encouraging the exchange of ideas and goods.^19^ Social capital has also been shown to be a more cost-effective agricultural intervention in overcoming social barriers to adaptation to climate change.^32^This was also evident in our study, where technical knowledge of cultivation techniques, such as ‘vertical cultivation’ named “Waga Kuluna,” has helped to promote sustainable gardening practices.

 Social capital aids in livestock development by facilitating the sharing of livestock knowledge, abilities, gifts, or loans and adopting technology.^33^ Similarly, linking social capital promoted technical support for curd and yogurt production and poultry farming in our case study. In addition to ensuring food security among members of the mothers’ support group, the exchange of fresh milk at a low cost promoted affordability and access to nourished foods. This transfer of technology has also been documented by other authors.^34,35^ Moreover, there is evidence that social capital directly correlates with the income of livestock farmers,^36^ and rebuilds their productive assets during a crisis.^37^

 Evidence shows that households with higher social capital are less likely to go hungry.^38^ We observed a phenomenon among mothers’ support groups where food wastage was minimized with knowledge sharing about food preservation techniques and self-employment generated through linking social capital. Furthermore, interaction among community members leads to the sharing of food and food products among communities, which in turn will strengthen food accessibility.^19^

 One of the mechanisms people in rural areas use as a buffer from economic and health crises is their kinship network, which is recognized as the foundation of social capital.^20^ The kinship network illustrates the social links and relationships that people have within their village with their extended families and relatives.^20^ The mothers’ support groups in the village reached into the existing kinship networks and benefitted from the relations, expertise, and resources available within the extended family through these extended family connections. This was prominently observed in selling harvests from home gardens among these kinship networks (bonding social capital). Economic development officers provided technical assistance for these activities for self-employment, thus inspiring linking social capital. These activities not only strengthened food accessibility^19^ but also entrepreneurship intentions^39^ in communities.

 Cognitive social capital is found to be positively associated with psychological health.^40^Similarly, stress and poor mental well-being have been identified among pregnant women living in neighborhoods with poor social capital.^41^ Furthermore, poor psychosocial factors are directly linked to poor nutrition.^42^ MSG improved psychosocial health promotion through norm creation and adoption of positive behaviors using bonding and bridging social capital, such as the adoption of norms such as the “Happiness Calendar” and “Buddi Kataya” (local savings scheme) in communities. The happiness calendar works by giving positive feedback to the family on the days the whole family was happy and without any psychosocial distress marked by the children of the family. The local saving schemes implemented by MSGs helped families save and invest in nutrition. Additionally, trust has been shown to influence health and happiness through social support and social networks (via MSG), which was shown in many places in our case study.^40^

 The implications of the findings of this study include a detailed understanding of how social capital promotes nutrition at the community level. Our study also contributes to the field of public health and health promotion by strengthening the evidence that social capital can be used for nutrition promotion during crises. Furthermore, it provides a crucial starting point for identifying areas where others might concentrate on strengthening social capital in community-based interventions. The transferability of our findings depends on the context in which this case study was conducted and the context of the population we are comparing. The findings may not be transferable to situations outside a crisis or to a very urban population.

 One of the limitations of this case study was that we did not use a triangulation methodology to gather qualitative data, which may have affected rigor. This may have affected the reliability of the data used in this case study. Because of the focus of our research and the availability of rich data from the annual review presentations of the MSG, we primarily relied on this single data source. Although triangulation could have provided additional perspectives, we mitigated this limitation by implementing rigorous analytical procedures, including thorough transcription and systematic thematic analysis, to ensure the validity of our findings. Furthermore, since there is considerable overlap between the dimensions of social capital, categorizing the data into different attributes may not have been optimal. Additionally, a case study was conducted with one mother’s support group representing each administrative district in the country. Therefore, this may not fully reflect the overall situation in all administrative districts.

## Conclusions and Recommendations

 This case study is one of the first studies in the region to compare how social capital can be strengthened to promote nutrition in communities via community-based approaches in economic crises. In summary, these findings add substantially to our understanding of how social capital (mainly bonding, bridging, and linking) can influence various aspects of nutrition promotion in communities in terms of awareness building, home gardening, livestock management, food waste reduction, economic support, and psychosocial support. Furthermore, these results provide a significant first step toward identifying activities that other community groups can focus on improving social capital. Further research is warranted to assess the impact of activities implemented within communities on the improvement of nutrition skills and practices, particularly in relation to social capital. Future studies should focus on measuring the effects of the interventions that have already been implemented and examining how they contribute to enhancing nutrition-related knowledge and behaviors within the community context. By investigating the bond between these activities and social capital, researchers can gain insights into how community engagement and social connections influence nutritional outcomes.

## Acknowledgments

 The authors would like to acknowledge all mothers’ support group members and health staff for their support in conducting the national review activity.

## Competing Interests

 None declared.

## Ethical Approval

 Not applicable.
